# Imaging and Diagnostic Challenges in a Patient With Refractory Hypoglycemia Caused by Insulinomas Related to Multiple Endocrine Neoplasia Type 1

**DOI:** 10.7759/cureus.8208

**Published:** 2020-05-20

**Authors:** Michael E Nance, Ritika Verma, Cory DeClue, Mark Reed, Tarang Patel

**Affiliations:** 1 Internal Medicine, University of Missouri, Columbia, USA; 2 Pulmonary, Critical Care, and Environmental Medicine, University of Missouri, Columbia, USA; 3 Internal Medicine, University of Missouri Health Care, Columbia, USA

**Keywords:** insulinoma, multiple endocrine neoplasia type 1, hypoglycemia, hyperparathyroidism, parathyroid gland adenoma, positive emission tomography, octreotide scan, ga-68 dotatate scan, neuroendocrine tumor

## Abstract

Insulinoma is a rare neuroendocrine tumor. It may occur sporadically or as part of the genetic tumor syndrome multiple endocrine neoplasia type 1 (MEN1). Diagnosis is challenging because of the small size of insulin producing tumors that lead to hyperinsulinemia. Advances in imaging modalities may provide more accurate diagnosis of primary tumors, metastasis, and tumor functional status. Advances allow for improved medical and surgical management with new tools for research of neuroendocrine tumors. Surgical excision of the primary tumor is often curative; however, insulinomas in MEN1 syndrome are often multifocal with a high rate of recurrence presenting unique challenges in management. Here, we present the case of a 34-year-old male with recurrent hypoglycemic episodes and hyperparathyroidism diagnosed with multiple pancreatic insulinomas secondary to MEN1. Furthermore, we provide a brief review of the literature and discuss the approach to diagnosis and management in patients with MEN1 syndrome and future areas of investigation.

## Introduction

Insulinomas are neuroendocrine tumors (NET) that arise from pluripotent stem cells in the pancreatic ductal or acinar epithelium with characteristics of insulin-secreting beta cells [1]. In functional insulinomas, insulin secretion causes hypoglycemic episodes, typically following periods of fasting or exercise. Neurological symptoms caused by brain glucose deprivation are the most common presentation and often mimic other neurological or psychiatric illnesses resulting in delayed diagnosis. The majority of insulinomas are small, frequently <1 cm, and curable with surgical resection if metastases are not present. Unlike sporadic cases, insulinomas may also be associated with genetic tumor syndromes, such as multiple endocrine neoplasia type 1 (MEN1). Insulinomas in MEN1 syndrome tend to be multifocal and more aggressive. Furthermore, the co-occurrence of additional tumors may further confound diagnosis and management. We present a 34-year-old man with a several year history of recurrent hypoglycemic episodes and hypercalcemia found to have an insulinoma secondary to genetically verified MEN1 syndrome.

## Case presentation

**Figure 1:**
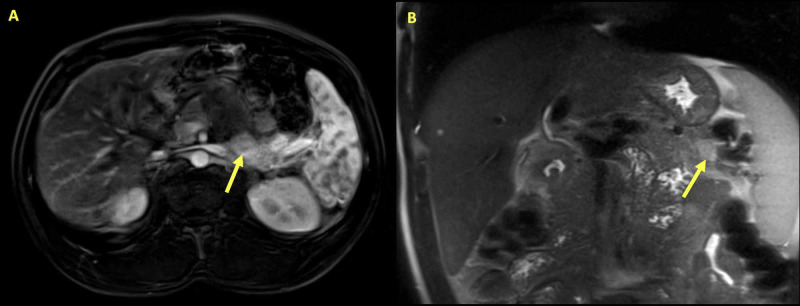
Abdominal Magnetic Resonance Imaging (A) T1-weighted axial section and (B) T1-weighted coronal section of abdominal magnetic resonance imaging with arrows highlighting a 2.8 cm x 1.3 cm area of variable enhancement and diffusion restriction within the pancreas, consistent with insulinoma.

A 34-year-old Hispanic male presented to the emergency room with an intermittent epigastric abdominal pain of one year. He endorsed a 10-pound weight loss due to decreased appetite and post-prandial nausea and vomiting. He did not take any medications regularly, and his family history was remarkable for a brother with Lynch syndrome. Five years prior, he had been diagnosed with primary hyperparathyroidism presenting with urolithiasis secondary to hypercalcemia. He also had a non-specific history of recurrent hypoglycemic episodes. More recently, he had episodic confusion for one to two weeks during which he could not recognize other people, familiar places, or even recall who he was. Multiple episodes occurred while working, which prompted his employer to send him to the emergency department. On exam, the patient was tachypneic and hypertensive with mild epigastric abdominal tenderness to palpation. There were no focal neurological deficits. Initial laboratory studies were remarkable for mild normocytic anemia, blood glucose 39 mg/dL, calcium 11.5 mg/dL, and phosphorus of 1.8 mg/dL. An intravenous (IV) infusion of 5% dextrose in half-normal saline was started for hypoglycemia. Subsequently, pamidronate along with zoledronic acid was administered for the hypercalcemia. With therapy, blood glucose improved to 107 mg/dL. After stabilization, the intact parathyroid hormone was measured at 180 pg/mg, which confirmed the presence of primary hyperparathyroidism. An ultrasound of the head and neck did not show evidence of parathyroid hyperplasia. Due to suspicion for insulinoma, fasting levels of insulin, c-peptide, and proinsulin were obtained. While random serum insulin and c-peptide were within normal limits (insulin: 6.0 µU/mL reference: 1-35 µU/mL, C-peptide: 0.903 nmol/L, reference: 0.34-2.33 nmol/L), proinsulin was elevated (63 pmol/L, reference: 3.6-22 pmol/L), which was suggestive of a diagnosis of insulinoma. The patient was started on diazoxide therapy, while a magnetic resonance imaging (MRI) of the abdomen was performed to identify potential NETs and possible metastases. The MRI of his abdomen revealed multiple pancreatic masses, the largest measuring 2.8 x 1.3 cm, with diffusion restriction and variable enhancement within the pancreas, consistent with insulinomas (Figure 1).

For confirmation of diagnosis, an esophagogastroduodenoscopy with endoscopic ultrasound was performed. Fine needle aspiration of three pancreatic lesions demonstrated pathology consistent with grade 1 pancreatic NET. Given the presence of hyperparathyroidism and pancreatic NET, MEN1 syndrome was suspected. The patient underwent genomic sequencing for menin mutation. Results of genetic testing demonstrated c.722G>A; p.Cys241Tyr variant in the MEN1 gene consistent with MEN1 syndrome. Screening tests for pituitary adenoma, including prolactin, thyroid-stimulating hormone (TSH), insulin-like growth factor-1 (IGF-1), adrenocorticotropic hormone (ACTH), follicle-stimulating hormone (FSH), and luteinizing hormone (LH) levels, were within normal limits. Therefore, an MRI of his brain was deferred. Surgical oncology was consulted for therapeutic options and recommended management with diazoxide and an octreotide scan for tumor staging.

**Figure 2:**
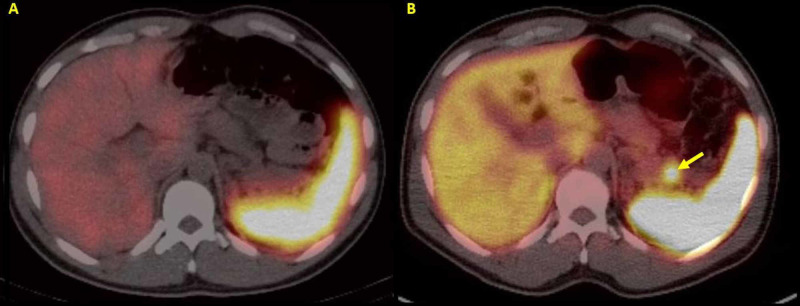
Abdominal Positron Emission Tomography (PET) Using Indium-111 Pentetreotide and Gallium-68 Dotatate (A) Coronal section of an abdominal PET scan using indium-111 pentetreotide (OctreoScan) demonstrating no focal uptake abnormalities, and (B) a coronal section of an abdominal PET scan completed using gallium-68 dotatate with the arrow demonstrating foci of radiotracer uptake in the pancreatic tail confirming the presence of neuroendocrine neoplastic foci.

An indium-111 pentetreotide (octreotide) scan was performed with imaging at 48 hours post-injection. It revealed no abnormal areas of uptake, including lesions previously identified on MRI (Figure 2A). Given conflicting imaging findings, a gallium-68 (68Ga) dotatate scan was performed to investigate a possible malignancy further, which demonstrated two foci of radiotracer uptake in the pancreatic tail confirming the neuroendocrine neoplastic foci (Figure 2B). Subsequently, he underwent successful distal pancreatectomy for definitive surgical management of his insulinomas. Surgical pathology demonstrated multifocal, grade 2, well-differentiated NET with vascular invasion. Tumor margins were negative with 0/12 lymph nodes positive for metastases. 

At follow-up three weeks post-surgery, the patient was enjoying an improved quality of life without any episodes of hypoglycemia or hypoglycemic symptoms. He reported checking his fasting blood glucose two times daily since his surgery with fasting blood glucose ranging from 90 to 120 mg/dL without insulin therapy. 

## Discussion

The first case of insulinoma was described in 1927 by Wilder and colleagues [2]. The patient had a pancreatic tumor from which an extract demonstrated insulin-like activity in a rabbit. Whipple described the classic criteria for diagnosis in the 1930s, which include (1) symptoms of hypoglycemia, (2) low plasma glucose at the time of symptoms, and (3) relief of symptoms when the glucose is corrected [3]. It was not until 1954 that Wermer described the initial case of MEN1 syndrome [4]. Since then our understanding of the history, presentation, and pathologic aspects of insulinoma and its association with the MEN1 syndrome has become more established. A review of cases of insulinoma from 1966 to 2005 found that approximately 90% of insulinomas are intrapancreatic, and occur as solitary tumors <2 cm in size. While only 10%-30% of insulinomas are associated with MEN1 syndrome, tumors in MEN1 syndrome are often multifocal, occur at younger ages, and have a higher risk of recurrence [5]. We have reported the case of a patient with recurrent hypoglycemic episodes presenting with neuroglycopenic symptoms responsive to glucose replacement. In addition to meeting Whipple's criteria, he had an elevated fasting proinsulin level consistent with insulinoma (Table 1). An initial abdominal MRI demonstrated multifocal pancreatic disease; however, his octreotide scan did not demonstrate any abnormal uptake. He was initially managed with diazoxide, a medication that suppresses insulin secretion by directly opening ATP-dependent potassium channels on the beta-cell membrane. Previous clinical reviews have reported adequate glycemic control using diazoxide in approximately 50% of patients [6]. Medical management is often necessary in the preoperative period or in the management of cases in which surgery is contraindicated. Surgical management offers the best chance for cure. An analysis of 46 cases of distal pancreatectomy demonstrated no recurrence when additional tumor foci were enucleated after 10 years [7]. Additionally, if surgical excision is possible, long-term survival is 88% at 10 years even in patients with MEN1 syndrome.

**Table 1:** The Differences in Various Serum Markers Amongst Etiologies of Hypoglycemia IGF: Insulin-Like Growth Factor

Diagnosis	Normal	Exogenous insulin	Insulinoma	Oral hypoglycemic agent	Insulin autoimmune syndrome	IGF	Not insulin (or IGF)-mediated
Symptoms ± signs	No	Yes	Yes	Yes	Yes	Yes	Yes
Glucose (mg/dL)	<55	<55	<55	<55	<55	<55	<55
Insulin (µU/mL)	<3	>>3	≥3	≥3	>>3	<3	<3
C-peptide (nmol/L)	<0.2	<0.2	≥0.2	≥0.2	>>0.2	<0.2	<0.2
Proinsulin (pmol/L)	<5	<5	≥5	≥5	>>5	<5	<5
Glucose increase after glucagon (mg/dL)	<25	>25	>25	>25	>25	>25	<25
Beta-hydroxybutyrate (mmol/L)	>2.7	≤2.7	≤2.7	≤2.7	≤2.7	≤2.7	>2.7
Insulin antibodies	–	+/–	–	–	–	–	–
Circulating oral hypoglycemic agent	No	No	No	Yes	No	No	No

The success of the surgical intervention relies heavily on accurate preoperative imaging and the ability to clearly localize the tumor. However, imaging insulinomas, specifically in MEN1 syndrome, is notoriously difficult due to their small size and often extrapancreatic locations. Somatostatin receptor (SSTR) scintigraphy with 111In-pentetreotide computed tomography (OctreoScan) is one method of detecting foregut NETs, but it fails to image 35%-50% of pancreatic NETs <1 cm in diameter [8]. Endoscopic ultrasound can detect tumors <0.5 cm, but this is an invasive test and not always available [9]. Alternatively, positron emission tomography (PET) using 68Ga dotatate-conjugated somatostatin analogs (SSAs) has been shown to increase sensitivity threefold when compared with OctreoScan in a study of 26 cases of MEN1 [10]. Clinicians should be aware that imaging modalities that rely on conjugated SSAs are dependent on expression of SSTRs on NETs. Of the five known SSTRs, only SSTR4 was consistently expressed amongst benign and malignant insulinomas [11]. This inconsistency may result in more false negatives and failure to localize non-SSTR expressing primary or metastatic lesions. These challenges were highlighted in this case as the multifocal lesions seen on MRI of the abdomen were not identified using OctreoScan and only two lesions in the pancreatic tail were visualized with the 68Ga dotatate PET.

As patients with MEN1 syndrome are more prone to multifocal disease and recurrence, the development and validation of novel therapeutics holds the potential to greatly benefit these patients. Peptide receptor radionucleotide therapy using lutetium-177 or yttrium-90 conjugated to an SSA has shown promise for NETs in patients without MEN1. One controlled trial of 177Lu dotatate in 229 patients with advanced midgut NET showed progression-free survival of 11% vs 65% at 20 weeks in favor of the radioisotope group [12]. These promising results may offer additional tools to clinicians in the future; however, any receptor-based therapeutics will likely be limited secondary to histological differences in tumors as observed with OctreoScan and 68Ga dotatate imaging.

## Conclusions

We present a patient with recurrent symptomatic hypoglycemic episodes, who was found to have multifocal insulinomas and MEN1. Clinicians should be aware of the diverse presentations of insulinoma in patients with MEN1 syndrome. Rigorous preoperative staging with imaging is critical to successful surgical management. Currently both OctreoScan and 68Ga dotatate PET scans are available imaging modalities; however, both have significant limitations when attempting to identify insulinomas. Lutetium-177 dotatate offers a potential area of receptor-based therapy that could be both diagnostic and therapeutic. However, more robust clinical trials will need to be conducted in order to establish the role of 177Lu dotatate in insulinomas related to MEN1 syndrome. 
